# High Throughput Mining and Characterization of Microsatellites from Common Carp Genome

**DOI:** 10.3390/ijms13089798

**Published:** 2012-08-06

**Authors:** Peifeng Ji, Yan Zhang, Chao Li, Zixia Zhao, Jian Wang, Jiongtang Li, Peng Xu, Xiaowen Sun

**Affiliations:** 1 The Centre for Applied Aquatic Genomics, Chinese Academy of Fishery Sciences, Beijing 100141, China; E-Mails: theonejpf@gmail.com (P.J.); zhangyan19780927@163.com (Y.Z.); zhaozx@cafs.ac.cn (Z.Z.); wangj@cafs.ac.cn (J.W.); lijt@cafs.ac.cn (J.L.); 2 Heilongjiang Fishery Research Institute, Chinese Academy of Fishery Sciences, Harbin 150070, China; E-Mail: lichao_8295@yahoo.cn

**Keywords:** microsatellites, common carp, genome, linkage map, BAC end sequences

## Abstract

In order to supply sufficient microsatellite loci for high-density linkage mapping, whole genome shotgun (WGS) sequences of the common carp (*Cyprinus carpio*) were assembled and surveyed for microsatellite identification. A total of 79,014 microsatellites were collected which were harbored in 68,827 distinct contig sequences. These microsatellites were characterized in the common carp genome. Information of all microsatellites, including previously published BAC-based microsatellites, was then stored in a MySQL database, and a web-based database interface (http://genomics.cafs.ac.cn/ssrdb) was built for public access and download. A total of 3,110 microsatellites, including 1,845 from WGS and 1,265 from BAC end sequences (BES), were tested and genotyped on a mapping family with 192 individuals. A total of 963 microsatellites markers were validated with polymorphism in the mapping family. They will soon be used for high-density linkage mapping with a vast number of polymorphic SNP markers.

## 1. Introduction

The common carp (*Cyprinus carpio*) is one of the most important aquaculture species in the world, accounting for nearly 14 percent of the total global freshwater aquaculture production in 2002, which has increased by an average global rate of 9.5 percent/year [1]. In China, the common carp is the third most cultured freshwater species with 2,200 kilotons annual production. Because of the importance of this species, molecular genetics and genomics studies of carps have been conducted productively in the past decade in the world for genetic improvement purpose [2–7]. To date, a large number of polymorphic genetic markers, including several hundreds of microsatellite and SNP markers, have been developed for genetic linkage mapping and marker-assisted selection in breeding programs [8–11]. Several linkage maps have been constructed based on polymorphic RAPD and/or microsatellite markers [3,8,12]. Quantitative trait loci (QTL) analysis has been conducted on various economic important traits including growth, cold-tolerance, muscle quality, amino acid content, *etc.* [13–15]. A Bacterial Artificial Chromosome (BAC) library has been constructed and a large number of BAC ends have been sequenced [16,17]. A BAC-based physical map has been constructed [18]. Most excitingly, a large-scale whole genome-sequencing project for the common carp was funded and initiated by multiple institutes in China.

Although rapid progress is being made, some key elements, including high-density linkage mapping and physical mapping, are still unavailable in order to create a comprehensive whole genome assembly for molecular breeding and other usages of the common carp and related species. Considering the relative large genome size (1,700 Mbp) [19], a high-density linkage map with at least several thousand genetic markers is necessary to serve as a backbone for both genome map construction and important trait location. High-density genetic mapping requires a large number of genetic markers as anchor points in order to localize and orientate genome contigs and scaffolds on the high density linkage map. Although high throughput SNP genotyping methods could amp vast numbers of SNPs in the high-density linkage map, microsatellite markers are still useful genetic markers due to the feasibility and flexibility, especially for some small laboratories on certain species without sufficient SNPs for high throughput genotyping. Sometimes, budget is be also the major concern for the usage of high throughput SNP genotyping. BAC-anchored microsatellite markers developed from BAC end sequences could serve as important tools for BAC-based physical map and linkage map integration [20,21].

With the common carp whole genome-sequencing project, large-scale whole genome shotgun (WGS) sequences and BAC end sequences (BES) [17] have been collected and analyzed. Microsatellite loci are easy to obtain from the WGS data and BES data. Working toward the construction of a high-density linkage map for the common carp, high throughput microsatellite screening and characterization have been conducted on common carp WGS and BAC end sequences. A large number of microsatellites were identified from both WGS and BES. All microsatellites have been collected in a web-based database for better access for the common carp research community. Then a number of microsatellite markers have been validated and genotyped in the mapping population.

## 2. Results and Discussion

### 2.1. Microsatellite Statistics in Common Carp Genome

A total of 4,484,155 whole genomic shotgun sequences generated by Roche Genome Sequencer FLX System were surveyed. After bioinformatic procedures, a total of 982,074 unique sequences including contigs and singletons were used for microsatellite screening. As showed in Table 1, a total of 79,014 microsatellites were identified from WGS data in this survey. A total of 68,827 distinct sequences were found to harbor microsatellites, revealing that around 7.01% of common carp shotgun sequences contain at least one microsatellite with two to six nucleotide motifs. Of these microsatellite-harboring sequences, 60,259 sequences harbor only one microsatellite, 7,269 sequences harbor two microsatellites and 1,299 sequences harbor three or more microsatellites. On average, there was at least one microsatellite per 3.88 kb in the surveyed common carp genome. A total of 24,898 sequences with flanking regions greater or equal to 50 bp on both sides were selected as they may have sufficient flanking region for future primer design.

Similarly, BAC-anchored microsatellites were identified from BES of the common carp, which had been reported in the previous publication [17]. Thus, the BES-derived microsatellites had been collected for marker development and database construction. As shown in Table 1, a total of 10,355 BES were found to contain microsatellites with 13,581 microsatellites from the 65,720 common carp BES. A total of 5,150 BAC-anchored microsatellite markers from 3,456 BES sequences were developed on those loci with sufficient flanking regions.

Up until the past decade, the widely used traditional method for microsatellite identification and marker development in aquaculture species was microsatellite-enriched library and Sanger sequencing, and it is still used by many laboratories. It may take several months to collect enough microsatellite markers for some applications, such as genetic mapping, population evaluation or pedigree analysis for a “novel” species. The next generation of sequencing technology provides us with a more effective method for microsatellite identification and marker development. We could easily collect more than a thousand microsatellite sequences from a “novel” genome in several weeks.

All microsatellites identified in this study were based on genomic DNA sequences. In order to assess the number of expressed genes associated with microsatellite loci, we performed a homologous search against a non-redundant (nr) protein database with the BLASTx program (e-value cutoff of e-5). The results showed that 9,771 expressed genes were associated with microsatellites in this study. Most likely, these microsatellites are physically close to coding sequences, e.g., introns of the genes. It is important to develop Type I markers for genetic analysis. Alternatively, Expression-Sequence-Tag (EST)-derived microsatellites could be identified from ESTs or transcriptome sequences for gene-associated SSR development. For instance, a total of 2,064 microsatellites had been identified from common carp transcriptome sequences with next-generation sequencing, providing an efficient method for microsatellite marker development [22].

### 2.2. Characterization of Microsatellites

**Figure 1:**
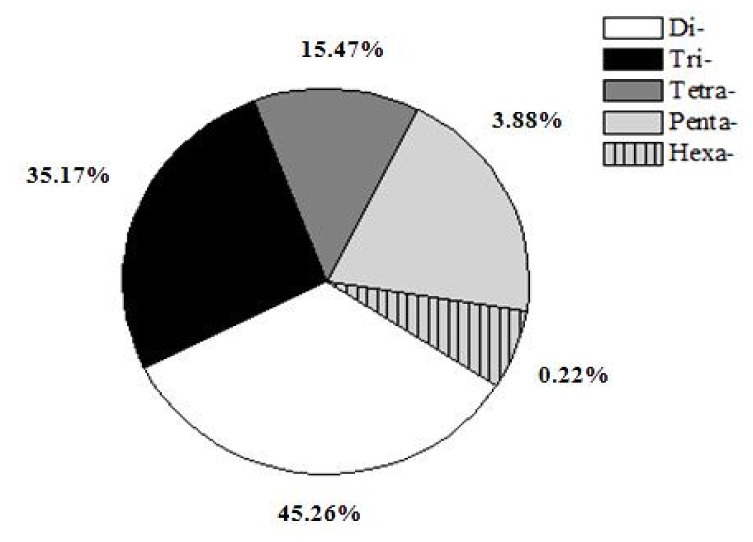
Distribution of five types of microsatellites.

**Figure 2:**
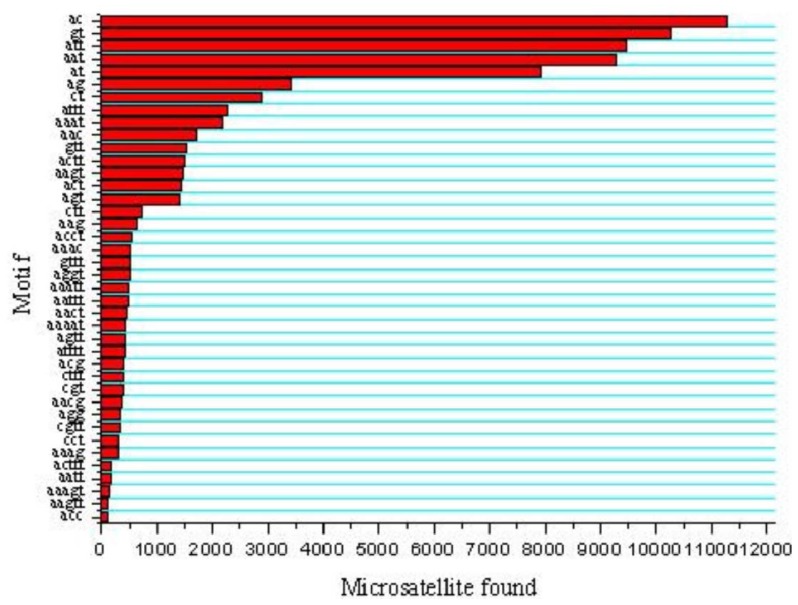
Distribution of major microsatellite types of the common carp.

Five types of microsatellites with di-, tri-, tetra-, penta-, hexa-nucleotide motifs were identified which were 35,760 (45.26%), 27,788 (35.17%), 12,222 (15.47%), 3,070 (3.88%) and 174 (0.22%), respectively (Figure 1). Of the di-nucleotide microsatellites, the most abundant motif types were AC (11,268; 31.51%), GT (10,256; 28.68%), AT (7,918; 22.14%), while AG (3,423; 9.57%) and CT (2,888; 8.08%) were much lower; and the CG type (7; 0.02%) was extremely rare (Figure 2). AC/GT type microsatellites were definitely to be the most dominant di-nucleotide microsatellites in the common carp genome (~60.2%), which is 9.5% more than in the catfish genome (50.7%) [21] and 6.4% more than in the tilapia genome (53.8%) [23]. This implies that the common carp might have more homologous recombination events that catfish and tilapia, as AC/GT has been reported to be able to affect homologous recombination in eukaryotes [24]. Of the tri-nucleotide microsatellites, the most abundant motif types were ATT/AAT (18,751/67.48%). All other tri-nucleotide motif types only occurred in less than 33%. A/T-rich motif, containing at least two A or T nucleotides, accounted for 95.16% of tri-nucleotide microsatellite motifs, while G/C-rich motifs, containing at least two G or C nucleotides, were only 4.84% of tri-nucleotide microsatellite motifs. Similarly, in tetra-, penta-, hexa-nucleotide microsatellites, the A/T rich microsatellites were also the vast majority compared with G/C-rich microsatellites. Evaluation and characterization of those BAC-anchored microsatellites has been conducted in a previous publication [17], which has a similar ratio and percentage to microsatellite statistics from WGS.

### 2.3. Database Construction for the Microsatellites

A web-based searchable database was constructed for the microsatellites and the sequences, which included microsatellites. Information in the database included microsatellites names, microsatellites containing sequence information, microsatellite motifs and locations, microsatellite primer names, primer sequences, and PCR conditions. The database was mounted on the website http://genomics.cafs.ac.cn/ssrdb for public access. In this database a total of 79,014 and 13,581 non-redundant microsatellites had been collected from WGS and BES, respectively, for future use in genetics and genomic research of the common carp and closely related Cyprinidae fishes, which have been previously reported [25,26].

### 2.4. Microsatellite Loci Validation

In order to validate our microsatellite discovery and present the usability of the database, a total of 3,110 microsatellite loci, including 1,845 from WGS and 1,265 from BES, were tested for the ongoing linkage mapping project of the common carp. A total of 963 microsatellite markers have been successfully validated as polymorphic markers in the mapping panel (Supplemental Table S1), which are exploited for linkage mapping and physical map/linkage map integration. More microsatellite markers will continually be developed based on the collected microsatellite dataset for these ongoing applications. With this validation, we hope to give more confidence to potential users in the community of the common carp microsatellite database.

## 3. Experimental Section

### 3.1. Whole Genome Shotgun Sequence Assembly

As part of the whole genome-sequencing project of the common carp, vast whole genome shotgun sequences were generated from a mitotic gynogenic double haploid common carp. In this particular research, a total of 4.48 million shotgun reads from Roche Genome Sequencer FLX System were used. The WGS data were collected and stored on the local server, and the software CLC Genomics Workbench (CLCbio, Aarhus, Denmark, 2011) was used to generate WGS contigs. Briefly, the adapters and low quality bases were removed by using the built-in adapter removal tools in CLC Genomics Workbench. After sequence trimming and clean-up, de novo assembly was performed with default parameters and the similarity was set as 0.8. The consensus sequences of contigs were used for microsatellite identification.

### 3.2. BAC End Sequences

As part of the whole genome-sequencing project of the common carp, a large scale BAC end sequencing project was conducted [17]. A total of 65,720 BES were collected, and used for BAC-anchored microsatellite identification and marker development.

### 3.3. Microsatellite Identification

A perl-based script Msatfinder V 2.0.9 [27] was used for microsatellite identification from both WGS contigs and BES. The mononucleotide repeats were ignored by modifying the configure file. The thresholds for di-, tri-, tetra-, penta-, hexa-nucleotide motifs were set as 8, 5, 5, 5 and 5, respectively. The search engine for finding perfect repeats in the program was set as “regex”.

### 3.4. Microsatellite Statistics and Characterization

The complete output from Msatfinder, containing the information of shotgun sequence ID, sequence lengths, microsatellite motif type, repeat units, microsatellite start and stop points, distance to both ends, was imported into the MySQL database on the local server for data refining and classification. Microsatellites containing genomic DNA sequences with flanking sequences greater or equal to 50 bp on either side of the microsatellites were collected for primer designing and marker development.

### 3.5. Microsatellite Database Construction

All identified microsatellites from WGS contigs or BES were tabulated into tab-delimited files and imported into the MySQL database, with the information of repeat_id (an unique name identifying this particular microsatellite), genome, genome_length, flank_length (the length of the flanking regions), repeat_plus_flank (Total length of the microsatellite and the flanks), start and stop (start and stop positions of the microsatellite within the genome), motif_units (the motif and number of repeat units, e.g., AT(6)), motif and motifrevcom (the motif (e.g., AT) and its reverse complement (e.g., TA)), footprint, repeat_units (number of repeat units), dist_from_right or left), pc_from_right, motif_type, primers (was it possible to make a primer for this microsatellite (1 = yes, 0 = no)), GC_content_flank, GC_content_repeat. The website of “Common Carp Microsatellite Database” was also constructed with a Apache and Tomcat platform.

### 3.6. Microsatellite Marker Validation and Genotyping

In order to test the microsatellite mining results and to provide enough microsatellite markers for the ongoing high-density linkage mapping, the microsatellites with sufficient flanking regions were selected for microsatellite marker development and validation in the F1 mapping panel with 192 full-sib individuals. Briefly, primers were designed for each unique sequence using Primer 5.0 (Premier Biosoft International: Palo Alto, CA, USA, 2007). A tailed primer protocol [28,29] with the following conditions was used to amplify the microsatellite alleles: 1× PCR buffer, 0.15 mM MgCl_2_, 0.2 mM each of the dNTPs, 0.15 pmol upper PCR primer, 6 pmol lower PCR primer, 0.15 pmol labeled tail primer, 0.5 units of DNA Taq polymerase (Fermentas, Vilnius, Lithuania), and 20 ng genomic DNA, in a total reaction volume of 15 μL. The tail primers were labeled with fluorescence on 5′ end. A touchdown PCR was performed with the following thermo profile: initial denaturation at 94 °C for 5 min, PCR amplification was carried out at 94 °C for 30 s, 56 °C for 45 s, and 72 °C for 45 s for 30 cycles as the first step, and at 94 °C for 30 s, 53 °C for 45 s, and 72 °C for 45 s for 10 cycles as the second step. A final extension at 72 °C for 10 min was included. The PCR products were analyzed on a 3130 XL Genetic Analyzer (Applied Biosystems, Foster City, CA, USA) and genotyped with LIZ-500 size standard (Applied Biosystems) using GeneMapper 4.0 software (Applied Biosystems).

The common carp was handled according to the Guidelines of Heilongjiang River Fishery Research Institute.

## 4. Conclusions

A total of 79,014 microsatellites were identified from the whole genome shotgun sequences of the common carp. A web-based microsatellite database was then constructed for public access and data sharing. A total of 92,595 microsatellite loci, including previously published 13,581 BAC-derived microsatellites, were archived. A small portion of these microsatellites were used for microsatellite marker development. Finally, 963 polymorphic markers were genotyped in the mapping population of the common carp. With our online database, any scientists in the common carp research community can search and download microsatellite data and design primers for their own research purpose. We will continue to collect and archive more microsatellites from Cyprinids as well as other aquaculture species either from public resources or from collaborators, and build the aquaculture microsatellite database for breeding and genetic improvement in aquaculture.

## Figures and Tables

**Table 1:** Statistics of microsatellites identified from whole genome shotgun sequences and BES.

	WGS	BES
WGS sequences surveyed	982,074 a	65,720 b
Base pairs surveyed (bp)	504,292,105 a	42,522,168 b
Microsatellite found	79,014 a	13,581 b
Sequences harboring microsatellites	68,827 a	10,355 b
Sequences harboring only one microsatellites	60,259 a	8,069 b
Sequences harboring two microsatellites	7,269 a	1,682 b
Sequences harboring three or more microsatellites	1,299 a	604 b
Microsatellite sequences with sufficient flanking regions on both sides *	24,898 a	5,150 b

a Data of WGS was new in the present study;

b Data of BES has already been shown in the earlier study [17];

* Number of distinct sequences with longer than 50 bp flanking sequences on both sides of microsatellite from WGS.
